# Women's empowerment and child nutrition: The role of intrinsic agency

**DOI:** 10.1016/j.ssmph.2019.100475

**Published:** 2019-11-20

**Authors:** Rebecca Jones, Regine Haardörfer, Usha Ramakrishnan, Kathryn M. Yount, Stephanie Miedema, Amy Webb Girard

**Affiliations:** a Nutrition and Health Sciences Program, Laney Graduate School, Emory University, USA; b Department of Behavioral Sciences and Health Education, Emory University, USA; c Hubert Department of Global Health, Emory University, USA; d Department of Sociology, Emory University, USA

**Keywords:** Women's empowerment, Nutritional status, Maternal and child health, East Africa

## Abstract

Women's empowerment is associated with improved child nutrition, and both underpin the achievement of multiple Sustainable Development Goals (SDGs). We examined pathways by which women's empowerment influences child nutritional status. We pooled nationally representative data from Demographic and Health Surveys (2011–2016) collected from married women with children aged 6–24 months in Ethiopia, Kenya, Rwanda, Tanzania, and Uganda (n = 13,780). We operationalized child nutritional status using anemia, height-for-age z-score (HAZ), and weight-for-age z-score (WHZ). We operationalized women's empowerment using a validated measure comprised of three latent domains: social/human assets (“assets”), intrinsic agency (attitudes about intimate partner violence), and instrumental agency (influence in household decision making). We used structural equation models with latent constructs to estimate hypothesized pathways from women's empowerment to child nutritional status with further mediation by maternal body mass index (BMI) and stratification by wealth. Women's empowerment domains were directly and positively associated with maternal BMI (estimate±SE: assets, 0.17 ± 0.03; intrinsic agency, 0.23 ± 0.03; instrumental agency, 0.03 ± 0.01). Maternal BMI was directly and positively associated with child HAZ (0.08 ± 0.04) and child WHZ (0.35 ± 0.03). Assets were indirectly associated with child HAZ and WHZ through intrinsic agency and maternal BMI. In the lowest wealth category, the direct effects from women's empowerment to child nutritional status were significant (assets and instrumental agency were associated with anemia; intrinsic agency associated with HAZ). In the highest wealth category, direct effects from women's empowerment on child nutritional status were significant (intrinsic and instrumental agency associated with WHZ). Improving women's empowerment, especially intrinsic agency, in East Africa could improve child nutrition directly and via improved maternal nutrition. These findings suggest that efforts to realize SDG 5 may have spillover effects on other SDGs. However, strategies to improve nutrition through empowerment approaches may need to also address household resource constraints.

## Highlights

• Empowerment domains associated differently with child nutrition in East Africa.
• Paths from empowerment to child nutrition differ by household wealth.
• Assets and Intrinsic agency more relevant for anthropometric status.

## 1 Introduction

In 2015, global leaders pledged to reduce chronic malnutrition in children younger than 5 years by 40 percent before the year 2025 (International Food Policy Research Institute, 2016). Despite global efforts, only three regions achieved at least a 50% decline in stunting from 1990 to 2015, (UNICEF, 2015). Although nutrition-specific interventions to improve maternal and child nutrition are efficacious (Bhutta et al., 2013; Ramakrishnan, Imhoff-Kunsch, & Martorell, 2014), these effects often are small in magnitude. Accelerating progress to achieve this new target requires not only expansion of evidence-based nutrition-specific strategies^1^ but greater prioritization and scale up of approaches that tackle the underlying determinants of child nutrition (Bhutta et al., 2013; Black et al., 2008, 2013; Ruel & Alderman, 2013).

Women's empowerment is one such underlying determinant of child nutrition, with substantial evidence that it positively influences child nutrition and growth (Carlson, Kordas, & Murray-Kolb, 2015; van den Bold, Quisumbing, & Gillespie, 2013). Scholars define women's empowerment as the ability of a woman to claim *enabling resources,* exercise voice and *agency,* and act on desires to transform her own life in contexts where this ability has been denied (Kabeer, 1999). In many societies, women are responsible for the procurement, preparation, and allocation of food and ultimately preserving food culture (Hodgson, 1999; Holtzman, 2002). As Holtzman describes, among the Samburu pastoralists of northern Kenya, “women are foremost constituted as food givers, responsible for providing sustenance to children and elders” (Holtzman p.1045). Thus, it is unsurprising that women's empowerment is associated with improved household and own dietary diversity (Amugsi, Lartey, Kimani, & Mberu, 2016;Galie et al., 2019 ; Malapit, Kadiyala, Quisumbing, Cunningham, & Tyagi, 2015; Yimer & Tadesse, 2015) and improved optimal infant and young-child feeding practices (Bose, 2011; Ickes, Heymsfield, Wright, & Baguma, 2017; Na, Jennings, Telegawker, & Ahmed, 2015; Shroff et al., 2011; Smith et al., 2002). Relative to disempowered women, more empowered women may have a greater influence on budgets allocated to household food procurement and how food is allocated in the household and greater agency in how they choose to feed their infants, contributing to improved diets and nutritional status. Integrating women's empowerment into social and behavioral strategies for improving nutrition may shift societal and household norms away from women's role as “last and least” in food prioritization (Carlson et al., 2015; Gittelsohn, Thapa, & Landman, 1997; Ruel & Alderman, 2013; Smith et al., 2002). Empowerment of women resulting in more equitable distributions of power within the household may contribute to prioritization of women's own nutrition and health, which may manifest in improved body mass index (BMI) and micronutrient status (Ramakrishnan, Grant, Goldenberg, Zongrone, & Martorell, 2012; Young, Nguyen, Addo, et al., 2015; Hambidge et al., 2019). This shift could enhance children's nutrition through direct biological transfer of nutrients in utero and in early infancy (Ackerson & Subramanian, 2008; Mason et al., 2014; Ramakrishnan et al., 2014; Shrimpton & Rokx, 2012; Wu, Imhoff-Kunsch, & Girard, 2012) and improved childcare capabilities throughout childhood (Engle, Menon, & Haddad, 1999; Matare, Mbuya, Pelto, Dickin, & Stoltzfus, 2015; Nussbaum, 2001).

Moreover, women's empowerment, despite differences in operationalization, has been associated with practices that indirectly affect child nutrition. Examples include practices that influence fertility, birth spacing, mental health, and health seeking e.g. (Abada & Tenkorang, 2012; Balk, 1994; Bloom, Wypij, & Das Gupta, 2001; Carlson et al., 2015; DeRose & Ezeh, 2005; Hadley, Patil, & Nahayo, 2010; Hou & Ma, 2013; James-Hawkins, Peters, VanderEnde, Bardin, & Yount, 2018a; Morgan & Niraula, 1995; Patel et al., 2006; Qadir, Khan, Medhin, & Prince, 2011; Rahman, 2012; Upadhyay & Hindin, 2005; Woldemicael & Tenkorang, 2010; Yount, Dijkerman, Zureick-Brown, & VanderEnde, 2014).

Despite these significant associations observed between women's empowerment and child nutrition, the pathways of influence and domains of women's empowerment most relevant for child nutrition remain due in large part to the heterogeneity in describing, defining, and operationalizing women's empowerment (Kishor & Subaiya, 2008; Pratley, 2016). Studies assessing associations between WE and child nutrition have operationalized this construct using myriad direct and indirect measures of women's empowerment. Moreover, scholars generally agree that women's empowerment is a process that operates in context-specific ways (Yount, Peterman, & Cheong, 2018). Heterogeneity in patriarchal family systems and the societal conditions that subordinate women (Kandiyoti, 1988) contributes to context specificity. For example, compared with South Asia and the Middle East, which historically are more classically patriarchal, women in East Africa participate to a greater extent in the household economy through income generation and often control low-revenue commodities such as vegetables, milk, or eggs (Dolan, 2001; Kandiyoti, 1988; Larsen & Hollos, 2003; Njuki, Kaaria, Chamunorwa, & Chiuri, 2011). Therefore, women in East Africa may hold greater autonomy in, for example, economic decision making than their South Asian and Middle Eastern counterparts. However, despite these opportunities for economic autonomy, persistent gender differentials in the value of what is controlled reflect gendered differences in intrahousehold power and influence household cooperation strategies, especially in rural agricultural households (Njuki et al., 2011). As a result, the process for women to become empowered and the domains most relevant for child nutrition may differ across contexts. Understanding the contextually relevant domains through which empowerment influences child nutrition is important for designing and implementing contextually appropriate policies and programs. Identifying those elements of empowerment that are relevant for child nutrition yet are stable or invariant across contexts may provide useful insights for monitoring empowerment and progress towards multiple Sustainable Development Goals SDGs related to both nutrition and gender.

Wealth is consistently associated with the quality of child diets and child nutritional status (Alderman, Haddad, Headey, & Smith, 2014; Alderman & Headey, 2018; Krishna et al., 2015). In keeping with Kabeer's framework, household wealth may be considered a resource for empowerment if it facilitates increased transfer of resources to women, as has been hypothesized in Kenya (Voronca, Walker, & Egede, 2018). However, as Hanmer and Klugman note (2016, pg 241), there exist “context-specific dimensions to and constraints, on choice—ncluding poverty and access to services—that shape the options that are open to people.” Therefore, there may be a threshold requirement for community, household, or individual wealth before women are able to act on their agency to achieve goals (Field, Jayachandran, & Pande, 2010; Habibov, Barrett, & Chernyak, 2017; Kabeer, 1999). In contrast, Kandiyoti argues that household poverty can weaken patriarchy because it compels women to go out of the home to work (Kandiyoti, 1988). Thus, in addition to examining the pathways by which women's empowerment is associated with child nutritional status, we explored the extent to which household wealth may confound or modify these pathways. Although we acknowledge the limitations in evaluating household wealth across five separate contexts, this measure is one of the few available to evaluate socioeconomic status within these samples. Furthermore, the Human Development Index (HDI) ranking for these five countries during our time period of study is between 0.463 and 0.590, which signifies a similar level of average achievement in key dimensions of human development across the countries.^2^

Based on this discussion, we propose three hypotheses for the cross-national evaluation of pathways of women's empowerment to child nutritional status in five countries in East Africa. First, in a pooled sample, we expect that women's empowerment, comprised of three latent factors (assets, intrinsic agency, and instrumental agency) will be associated with child nutritional status. Second, we expect that this relationship is mediated by women's nutritional status, measured using BMI. We acknowledge that the window for maternal nutrition to influence child nutrition may be narrow; however, this window may be wider than previously thought and may include preconception, with the more recent emphasis on preconception nutrition (Ramakrishan, Grant; Goldenberg et al., 2012; Young, Nguyen, Addo, et al., 2015; Hambidge et al., 2019). Furthermore, the implications are broader, given the importance of direct nutritional transfer and the impact of childcare capabilities. Finally, we evaluate the relationship of household wealth in confounding or modifying these proposed pathways. Evidence in support of these hypotheses would provide a theoretically informed, empirical example for using a subset of standard, cross-national survey items in multicountry assessments of the consequences of women's empowerment, in alignment with SDG 5.

## 2 Materials and methods

### 2.1 Framework and rationale

**Fig. 1:**
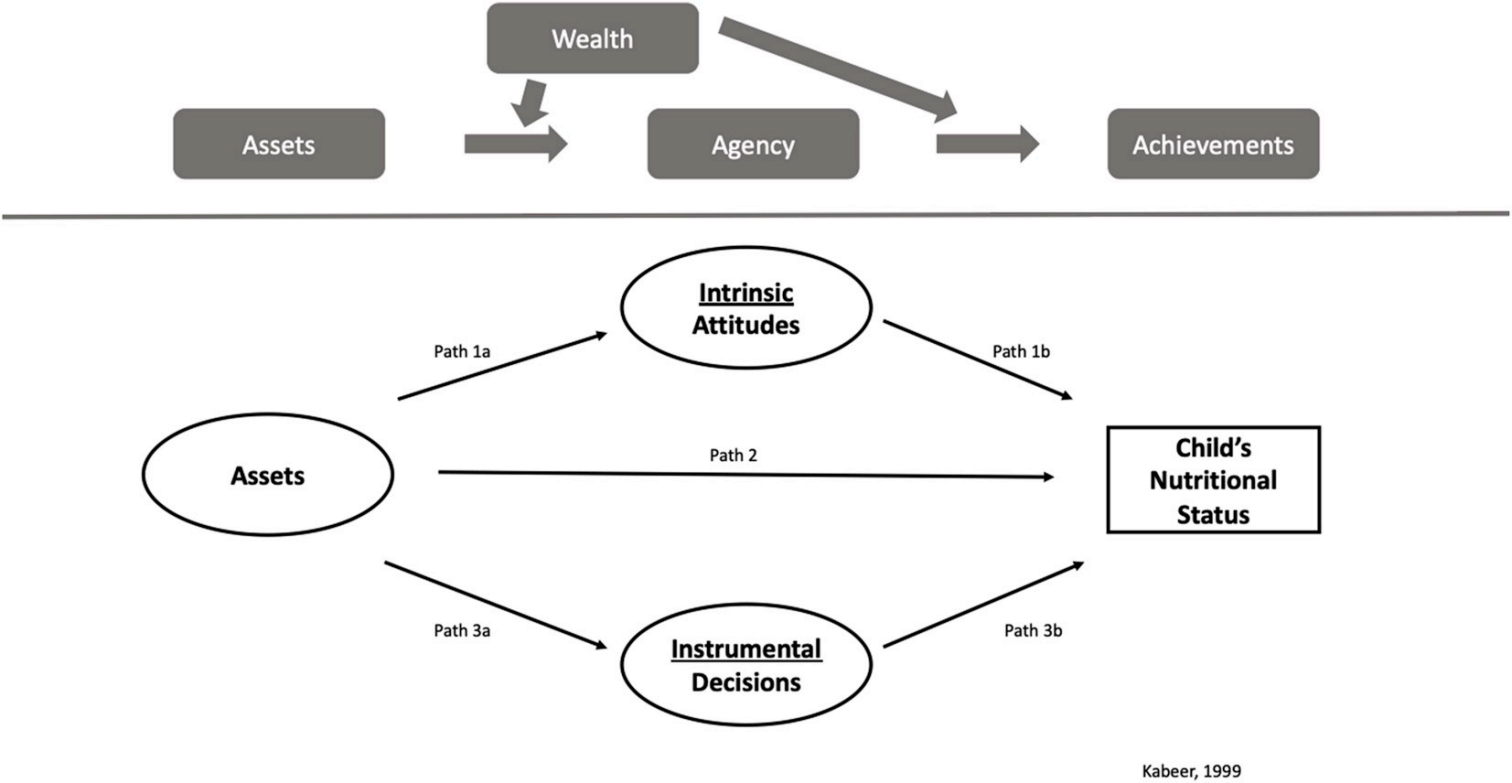
Conceptual tested path models from Women's empowerment to Children's nutritional status.

We adapted the framework proposed by Kabeer (1999) to depict enabling human and social assets as well as selected dimensions of women's agency as determinants of their achievements, in this case child nutritional status (Fig. 1). Using this adapted framework, we developed and psychometrically validated a three-dimensional model for women's empowerment in East Africa (Miedema, Haardörfer, Girard, & Yount, 2018) that was measurement invariant across five countries in East Africa: Kenya, Tanzania, Uganda, Rwanda, and Ethiopia. Although geographically contiguous and often assumed to be culturally homogenous, these five countries differ substantially in their historical trajectories, political systems, and sociocultural contexts, all of which influence gender norms and empowerment processes (Miedema et al., 2018). The lack of research on women's empowerment in East Africa relative to other regions and the heterogeneity in gender dynamics across this region provide strong rationale for testing an invariant model of women's empowerment across this region (Miedema et al., 2018) and assessing associations with child nutrition. The women's empowerment model developed through this process consisted of women's human and social assets, intrinsic agency to reject violence against women, and instrumental agency in household decisions. To fill this gap, we used a women's empowerment measure previously developed and validated by our team to quantify the influence of different empowerment domains on child anemia and anthropometry in five East African countries and the role of women's nutritional status as a mediator or confounder of this relationship. Height-for-age z-score (HAZ) is a measure of chronic undernutrition and captures linear growth in utero and early childhood (De Onis et al., 2007; WHO Multicentre Growth Reference Study Group, 2006). HAZ is among the preferred indicators of childhood nutrition and is a strong predictor of human capital (Victora et al., 2008). Weight-for-age z-score (WHZ) is an indicator of acute malnutrition. Anemia, measured using blood hemoglobin, is used as a proxy for iron deficiency in large-scale surveys. We hypothesized that each of these three domains of women's empowerment would be positively associated with child nutritional status and hemoglobin (defined as achievements).

### 2.2 Data resources and study population

We conducted a secondary analysis of data from the most recent Demographic and Health Surveys (DHSs) (2011–2016) for married women with children aged 6–24 months in Ethiopia, Kenya, Rwanda, Tanzania, and Uganda (n = 13,780). DHSs are nationally representative, cross-sectional surveys conducted in low- and middle-income countries since 1985 (ICF International, 2012b) and typically are based on a stratified, multistage probability sample design (Rutstein & Rojas, 2006). DHSs routinely include data on health and nutritional status based on anthropometric measurements and blood hemoglobin levels for women and children. Women were included in the present analysis if they were aged 15–49 years, nonpregnant, married or living with a partner at the time of the survey, and had a child aged 6–24 months with anthropometry measurements. (The sample size for each country was as follows: Ethiopia, n = 2664; Kenya, n = 4918; Rwanda, n = 1925; Tanzania, n = 2490; and Uganda, n = 1783). Overall, response rates for women eligible to take part in the DHS ranged from 93.8% to 99.1% across the DHSs in this study.

### 2.3 Variables

Three indicators of child's nutritional status were used in these analyses: HAZ, WHZ, and anemia status. We estimated children's nutritional status (HAZ and WHZ) using the World Health Organization (WHO) Child Growth Standard. Following altitude adjustment of hemoglobin, anemia status was categorized as either anemic or not anemic based on DHS cutoffs (<11.0 g/dL) (ICF International, 2012a; Pullum, Collison, Namaste, & Garrett, 2017). The 2014 Kenya DHS did not collect data on hemoglobin.

We operationalized women's empowerment using three latent domains—enabling resources, intrinsic agency and instrumental agency—identified through exploratory and confirmatory factor analysis in random split-half samples (Miedema et al., 2018). The invariance of these domains across countries was then examined through multigroup confirmatory factor analysis. The final three latent domains included three items focused on human and social assets that related to women's enabling resources (“assets”) and reflected levels of women's reproductive and marital power over their lifetime. These included ages at first sex, first cohabitation, and first birth.^3^ The latent domain of intrinsic agency captured whether women's expression of gender attitudes rejected or reflected normative beliefs of gendered violence (Yount, Krause & VanderEnde, 2016); it included five items related to women's attitudes about justification of wife beating under various conditions. The latent domain of instrumental agency measured women's ability to exercise decision making in the household. This domain included four items related to influence in decisions about large purchases, visiting family and friends, respondent's earnings, and respondent's health. The response options were respondent, respondent's partner/husband, both the respondent and her partner/husband, or someone else. Responses were coded 1 for women who reported sole or joint decision making with husband and 0 otherwise. More specifics about each WE domain and individual indicators are found in the Appendix (Supplemental Table 1).

We previously identified positive and direct associations between domains of women's empowerment and women's nutritional status (Jones et al., 2019). Thus, we treated maternal nutritional status as a mediator measured as BMI (defined as weight in kg/[height in m]^2^).

We used the DHS household wealth index as a measure of socioeconomic status. The DHS household wealth index is a composite measure of a household's cumulative living standard. The wealth index is calculated using data on a household's ownership of selected assets, such as televisions and bicycles; materials used for housing construction; and types of water access and sanitation facilities (Macro International Inc, 2006). The index is generated through principle components analysis, and the derived score from the first component ranks households on a continuous scale of relative wealth. The DHS then separates all interviewed households into five wealth quintiles to allow for the ability to compare the influence of wealth. For ease of interpretation, we recalculated these quintiles into three categories—low, middle, and high household wealth—to evaluate differences in hypothesized paths across household wealth groups and ran analyses stratified on these categories. We acknowledge that based on Kabeer's framing, wealth can also be a resource for empowerment; however, because of the cross-sectional nature of the data, we are restricted to evaluating wealth as a modifier. The overarching aim of this study was to evaluate a previously created measurement-invariant three-factor construct of women's empowerment and its associations with health outcomes (Miedema et al., 2018). Given this focus, we evaluated household wealth in the pooled sample. To strengthen our results, we further evaluated individual country paths by controlling first for household wealth and then stratifying by household wealth (Appendix).

### 2.4 Statistical analysis

We calculated means and standard deviation for all women's empowerment and health items for the overall sample (Table 1, Table 2) as well as by individual country (Supplemental Table 2). The relative frequencies of all variables were estimated to assess completeness and distributions. Spearman's rho correlations (Kline, 1998) were estimated to measure bivariate associations between items. For continuous variables, correlations were also assessed. STATA 14.1 software (College Station, Texas) was used to run descriptive analyses. Data were transferred to Mplus 8 statistical software (Los Angeles, California) for all model building, path analyses, and estimation.

**Table 1:** Selected characteristics of East African women and children sampled by demographic health surveys (2011–2016) (n = 13,780).

Variable	Ethiopia 2011		Kenya 2014		Rwanda 2014–2015		Tanzania 2015–2016		Uganda 2011	
	(n = 2664)		(n = 4918)		(n = 1925)		(n = 2490)		(n = 1783)	
	Mean /%	SD	Mean /%	SD	Mean /%	SD	Mean /%	SD	Mean /%	SD
Mother										
Age	28.1	6.37	28.0	6.24	30.1	5.93	28.5	6.90	28.0	6.49
Education										
No education	71%		24%		13%		24%		16%	
Primary	25%		51%		74%		58%		62%	
Secondary	3%		19%		11%		17%		17%	
Higher	2%		7%		3%		1%		5%	
Parity	2.3	1.16	2.4	1.25	2.2	0.78	2.6	0.82	3.1	0.51
Urban	18%		32%		22%		22%		20%	
Body mass index										
Underweight	26%		13%		4%		8%		14%	
Normal weight	68%		61%		70%		70%		71%	
Overweight	5%		18%		21%		15%		11%	
Obese	1%		7%		5%		7%		4%	
Household wealth										
Low	48%		56%		45%		43%		47%	
Middle	16%		17%		18%		19%		18%	
High	36%		27%		36%		38%		36%	
Child										
Male	50%		51%		50%		49%		50%	

Note.

^a^ Abbreviation: SD, standard deviation.

**Table 2:** Women's Empowerment, BMI, and, Child Nutritional Status of East African Women and Children Sampled by Demographic Health Surveys (2011–2016) (n = 13,780).

**Variable**	Low		Middle		High	
	(n = 9210)		(n = 3068)		(n = 5800)	
	Mean /%	SD	Mean /%	SD	Mean /%	SD
**Domain: Human and Social Assets (Assets)**						
Age at first sex	16.5	3.15	17.0	3.25	18.1	3.65
Age at first cohabitation	17.6	3.42	18.2	3.54	19.4	4.02
Age at first birth	18.8	3.44	19.2	3.21	20.5	3.87
**Domain: Intrinsic Agency**						
Goes out without spouse	50%		57%		68%	
If neglects child	43%		49%		61%	
If argues with husband	51%		56%		69%	
If refuses sex	57%		63%		74%	
If burns food	66%		70%		80%	
**Domain: Instrumental Agency**						
For respondent's earnings	83%		88%		92%	
For respondent's health	68%		73%		75%	
For large purchases	52%		59%		61%	
For family/friend's visits	63%		68%		73%	
**Mediator:**						
Women's BMI	21.0	3.01	21.7	1.95	23.5	1.74
**Outcomes: Child Nutritional Status**						
Height-for-age z-Score	−1.4	1.78	−1.3	1.34	−1.0	1.64
Weight-for-age z-Score	−0.4	1.15	−0.3	1.21	−0.1	1.18
Anemia status	39%		43%		45%	

Note.

^a^ Abbreviation: SD, standard deviation.

^b^ Anemia status excluding Kenya (n = 4918).

^c^ In intrinsic agency, the proportion represents the women who disagree with wife-beating for various reasons listed.

^d^ In instrumental agency, the proportion represents the women who reported sole or joint decision making.

^e^ A full outline of the survey questions used to create the women's empowerment domains are in the Appendix, Table 1.

We used structural equation models (SEM) with latent constructs to estimate the strength of hypothesized pathways from enabling resources to child nutritional status, directly and through women's intrinsic and instrumental agency. We present models with standardized path coefficients using weighted least squares with mean and variance-adjusted estimation for categorical data, and standardized path coefficients that are less than |0.05| were considered trivial (Hatcher, 1994; Muthén & Muthén, 2001). Indirect effects were considered significant based on bootstrapping methods (Bollen & Stine, 1990). Each test was resampled 1000 times, and an accelerated 95% confidence interval (95% CI) was determined. When the 95% CI did not include zero, the indirect association was significant. The hypothesized model was first evaluated for all five countries individually (women's empowerment to child nutritional status) and adjusted for household wealth. Country-level samples were then merged and used for the full five-country path analysis. The model accounted for country-level sample clusters, using the DHS cluster variable by country. Next, we evaluated the five-country model mediated by maternal BMI for outcomes HAZ and WHZ. Finally, we evaluated a multigroup model stratified by household wealth (low, middle, and high) for the full five-country sample mediated by maternal BMI. We further evaluated these stratified household wealth models by each individual country and saw no large differences in trends (Supplemental Fig. 3) Of note, the path analysis for Rwanda was unique with respect to associations between empowerment domain and indicators of child nutritional status (Supplemental Fig. 4). A sensitivity analysis was run for the full country model, with wealth stratification excluding the Rwandan sample (n = 11,855). There were some differences between country models, specifically with regard to associations between empowerment domains; however, because of the theoretical reasoning and the focus on empowerment's association with nutritional status, we present full five-country results. Because of low correlations between child nutritional outcomes and maternal age in all five countries, we did not control for age.

Our interpretations of the adequacy of model fit are based on theoretical interpretation and three measures of goodness-of-fit: the Comparative Fit Index (CFI), Tucker Lewis Index (TLI), and root mean square error of approximation (RMSEA) (Kline, 1998). Acceptable threshold levels for fit indices were CFI greater than 0.95, TLI greater than 0.95, and RMSEA less than 0.6 (Hooper, Coughlan, & Mullen, 2008). Chi-square fit indices consider acceptable threshold level as low χ^2^ relative to degrees of freedom and a nonsignificant *P* value (Hooper et al., 2008). However, because of the sample sizes required for such models, these fit indices are rarely informative and we do not report them (Bandalos & Finney, 2010). All SEM analyses were conducted using Mplus software, and direct associations, indirect associations, and standard errors were generated for each pathway.

## 3 Results

### 3.1 Sample characteristics

Table 1 shows selected demographic characteristics for women with children aged 6–24 months by country. Not all variables in Table 1 are used for further analyses but rather for a picture of the sample within each country. Mean age of the women ranged between 28 years (Kenya, Uganda) and 30 years (Rwanda). Mean education (in years) of the women ranged from 4.7 to 6.4 years, with the exception of Ethiopia, in which the mean education was 1.4 years. On average, women had had between 2 and 3 children. The percentage of women living in an urban area ranged from 18% in Ethiopia to 32% in Kenya.

Table 2 shows selected characteristics for women in the full sample by household wealth categories (n = 13,780). The measures of enabling resources, mean ages at first sex, first cohabitation, and first birth, increased with increasing household wealth. With regard to the measures of intrinsic agency, the percentage of respondents who did not justify wife-beating under certain conditions varied by condition and household wealth category; 43% of respondents in the low wealth category did not justify wife beating if a woman neglected her children compared with 49% and 61% in the middle and high wealth categories, respectively. If a woman burned food, 66%–80% of all respondents did not believe this was grounds for abuse. In relation to instrumental agency, 84% or more of respondents were responsible for decisions about their own incomes. For the other three items, the proportion of women reporting sole or joint decision making increased with increasing household wealth category (*P* < .05).

The mean BMI of women was in the normal range across all wealth categories, ranging from 21.0 ± 3.1 kg/m^2^ in the low category to 23.5 ± 1.7 kg/m^2^ in the high category. Across wealth categories, mean HAZ and WHZ scores ranged from −1.0 to −1.4 and from −0.1 to −0.4, respectively. The prevalence of anemia ranged from 39% to 45%.

### 3.2 Reduced-form latent structural equation model

**Fig. 2:**
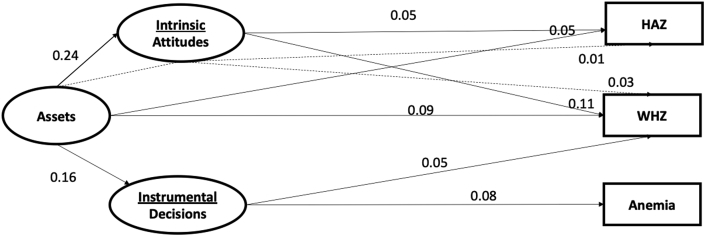
Diagram of direct and indirect standardized path coefficients from Women's empowerment to Children's nutritional status (N = 13,780). Note. ^*a*^ Model fit statistics: df = 78; CFI = 0.995; TLI: 0.993; RMSEA: 0.004 (0.001,0.006). ^b^ Significant paths based upon 95% confidence intervals. ^c^ All nonsignificant paths were dropped for simplicity. ^d^ Anemia model excludes Kenya (n = 4918). ^e^ Abbreviations: HAZ, height-for-age z-score; WHZ, weight-for-age z-score. df, degrees of freedom; CFI, comparative fit index; TLI, tucker lewis index; RMSEA, root-mean square error of approximation.

For our reduced-form five country model (Fig. 2), the standardized direct association of intrinsic agency with child HAZ was 0.05 (95% CI, 0.01, 0.09). Each unit increase in intrinsic agency was associated with an increase of 0.08 in HAZ (Table 3). Intrinsic agency and assets also were directly associated with WHZ (0.11 [0.03, 0.18] and 0.09 [0.01,0.17], respectively). Each unit increase in intrinsic agency was associated with an increase of 0.09 in WHZ. Instrumental agency was directly associated with WHZ (0.05 [0.02, 0.08]) and Anemia (0.08 [0.05, 0.11]). The indirect associations between assets and HAZ (0.01 [0.003, 0.02]) and WHZ (0.03 [0.007, 0.05]) through intrinsic agency were significant.

**Table 3:** Nonstandardized Path Coefficients for Direct, Indirect (where applicable), and Total Effects From Women's Empowerment to Children's Nutritional Status (N = 13,780).

Path and Dependent Outcomes	Latent Predictors	Direct Effect (β)	Indirect Effect (β)	Total Effect
Path 1: Intrinsic Agency	Assets	0.22 (0.20, 0.25)		
Path 1: HAZ	Intrinsic Agency	0.05 (0.01, 0.08)		
Through intrinsic agency	Assets		0.01 (0.003, 0.02)	0.06 (0.01,0.07)
Path 1: WHZ	Intrinsic Agency	0.09 (0.07, 0.12)		
Through intrinsic agency	Assets		0.02 (0.015,0.03)	0.11 (0.04,0.13)
Path 1: Anemia	Intrinsic Agency	0.01 (−0.02, 0.04)		
Through intrinsic agency	Assets		0.002 (−0.005, 0.009)	0.01 (−0.02,0.05)
Path 2: HAZ	Assets	0.04 (0.01, 0.07)		
Path 2: WHZ	Assets	0.08 (0.06, 0.11)		
Path 2: Anemia	Assets	0.06 (0.03, 0.08)		
Path 3: Instrumental Agency	Assets	0.14 (0.12, 0.16)		
Path 3: HAZ	Instrumental Agency	−0.02 (−0.06, 0.01)		
Through instrumental agency	Assets		−0.003 (−0.008, 0.001)	−0.02 (−0.05,0.01)
Path 3: WHZ	Instrumental agency	−0.01 (−0.03, 0.02)		
Through instrumental agency	Assets		0.000 (−0.004, 0.003)	−0.003 (−0.03,0.03)
Path 3: Anemia	Instrumental agency	0.09 (0.06, 0.13)		
Through instrumental agency	Assets		0.01 (0.007, 0.02)	0.10 (0.05,0.14)

Note.

^*a*^ Model fit statistics: df = 78; CFI = 0.995; TLI: 0.993; RMSEA: 0.004 (0.001,0.006).

^b^ 95% confidence intervals based on bootstrapping methods.

^d^ Anemia model excludes Kenya (n = 4918).

^e^ Abbreviations: HAZ, height-for-age z-score; WHZ, weight-for-age z-score. df, degrees of freedom; CFI, comparative fit index; TLI, tucker lewis index; RMSEA, root-mean square error of approximation.

When evaluating the associations between empowerment domains across countries, the standardized associations between assets and intrinsic agency (0.24 [0.19, 0.28]) and instrumental agency (0.16 [0.07, 0.25]) were significant (Fig. 2). Country-specific path analyses differed somewhat in the strengths of associations and some pathways (See Supplemental Fig. 1). For example, in Rwanda, assets were not associated with either intrinsic or instrumental agency, while in Kenya, assets were associated with both intrinsic agency (0.22 [0.17, 0.25]) and instrumental agency (0.13 [0.09, 0.16]).

### 3.4 Mediation model with maternal BMI

**Fig. 3:**
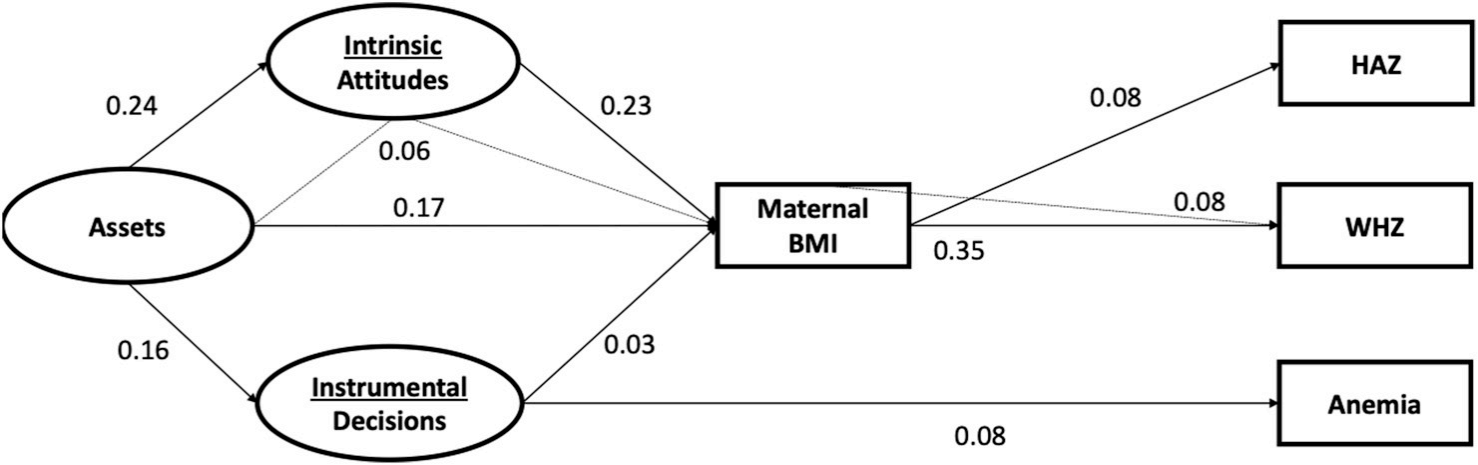
Diagram of direct and indirect standardized path coefficients from Women's empowerment to Children's nutritional status mediated by Women's BMI (N = 13,780). Note. ^*a*^ Model fit statistics: df = 88; CFI = 0.994; TLI: 0.991; RMSEA: 0.004 (0.002,0.006). ^b^ Significant paths based upon 95% confidence intervals. ^c^ All nonsignificant paths were dropped for simplicity. ^d^ Anemia model excludes Kenya (n = 4918). ^e^ Abbreviations: BMI, body mass index; HAZ, height-for-age z-score; WHZ, weight-for-age z-score. df, degrees of freedom; CFI, comparative fit index; TLI, tucker lewis index; RMSEA, root-mean square error of approximation.

When accounting for mediation by maternal BMI, assets were no longer directly associated with any child nutritional outcomes (Fig. 3). Instrumental agency was the only direct association which stayed significant after incorporation of maternal BMI (0.08 [0.05, 0.10]). All three domains of women's empowerment were significantly associated with maternal BMI, with attitudes being the strongest estimate (assets: 0.17 [0.14, 0.20]; intrinsic agency: 0.23 [0.20, 0.26]; instrumental agency: 0.03 [0.01, 0.05]).

Women's BMI was positively and directly associated with WHZ (0.35 [0.26, 0.43]) and child HAZ (0.08 [0.05, 0.10]). The indirect path from assets to intrinsic agency to maternal BMI was significant (0.06 [0.03, 0.080]). The extension of the indirect path from intrinsic agency through women's BMI to WHZ was significant (0.08 [0.04, 0.11]).

### 3.4 Reduced form latent structural equation model by household wealth

**Fig. 4:**
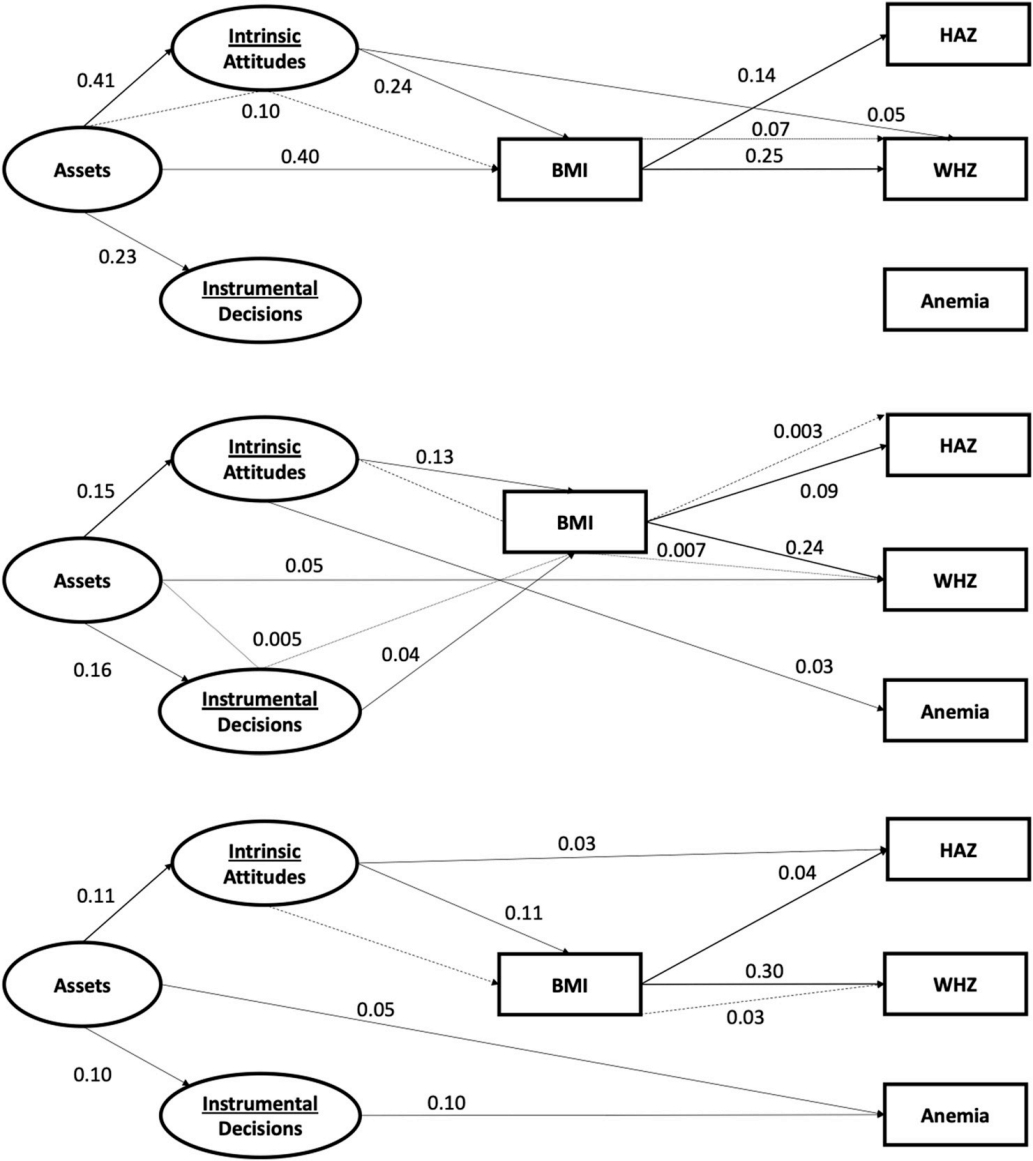
Diagram of direct and indirect standardized path coefficients from Women's empowerment to Children's nutritional status mediated by Women's BMI, by wealth groups (N = 13,780). A. Low. B. Middle. C. High. Note. ^*a*^ Model fit statistics: df = 288; CFI = 0.990; TLI: 0.988; RMSEA: 0.008 (0.006,0.009). ^b^ Significant paths based upon 95% confidence intervals. ^c^ All nonsignificant paths were dropped for simplicity. ^c^ Anemia model excludes Kenya (n = 4918). ^d^ Wealth categories based on Demographic and Health Survey household wealth index. ^e^ Abbreviations: BMI, body mass index; HAZ, height-for-age z-score; WHZ, weight-for-age z-score. df, degrees of freedom; CFI, comparative fit index; TLI, tucker lewis index; RMSEA, root-mean square error of approximation.

Paths from assets to intrinsic and to instrumental agency were higher in magnitude with greater household wealth, particularly from middle to high wealth category (Fig. 4). In examining associations between domains of women's empowerment and CNS, we noted that domains of women's empowerment operated differently depending on wealth categories.

Assets were directly associated with anemia in the lowest wealth category (0.05 [0.008, 0.09]) and with WHZ in the middle wealth category (0.05 [0.06, 0.15]). Intrinsic agency had a direct positive association with child HAZ in the low wealth group (0.03 [0.01, 0.05]), with anemia in the middle wealth group (0.03 [0.01, 0.05]), and with WHZ in the high wealth group (0.05 [0.03, 0.18]). Instrumental agency was associated only with anemia status in the lowest household wealth group (0.10 [0.08, 0.13]).

In the low wealth group, intrinsic agency were associated with maternal BMI (0.11 [0.08, 0.14]). BMI was subsequently associated with HAZ (0.04 (0.02, 0.06]) and WHZ (0.30 [0.27, 0.33]). In the middle wealth group, intrinsic agency (0.13 [0.10, 0.15]) and instrumental agency (0.04 [0.02, 0.06]) were directly associated with maternal BMI. In the high wealth group, assets (0.40 [0.37, 0.42] and intrinsic agency (0.24 [0.21, 0.26]) were both directly associated with maternal BMI.

Indirect effects differed by household wealth, with the majority of effects coming through intrinsic agency. The path from attitudes through BMI to WHZ was significant in the low wealth group (0.03 [0.01, 0.06]). This same path stayed consistent in the middle (0.007 [0.004, 0.01]) and high wealth groups (0.07 [0.04, 0.09]). An indirect path from intrinsic agency through BMI to HAZ was significant in the middle wealth group (0.003 [0.002, 0.005]). Goodness-of-fit statistics across models exceeded the threshold.

## 4 Discussion

Using a three-domain construct of women's empowerment grounded in Kabeer's assets, agency, and achievements framework, we observed that empowerment domains were associated differently with child nutrition in East Africa. Two general patterns emerged from the findings. First, the latent domain for human and social assets, represented as age at pivotal life events, and that for intrinsic agency, represented as attitudes to intimate partner violence, appeared more relevant for anthropometric status, while women's instrumental agency, measured as participation in household decision making was more relevant for anemia. Associations between empowerment domains and nutritional outcomes differed by wealth categories. For anthropometric status, but not anemia, the pathways from the women's empowerment domains were mediated through their effects on maternal BMI.

Prior research documented similar associations between maternal age at pivotal life events (the domain of assets), attitudes about intimate partner violence (IPV) (the domain of intrinsic agency), and indicators of child stunting (HAZ) and wasting (WHZ) (Carlson et al., 2015). Our use of a path analysis enabled deeper understanding of how these domains function. We observed that when maternal BMI was included as a mediator of the relationship, direct effects diminished and were replaced by pathways that operated through maternal BMI. This finding has several potential explanations and implications. First, women's empowerment serves as a foundational path to improve child nutrition. These improvements may derive from women's increased agency to ensure adequate food and care not only for their child but also for themselves (Richards et al., 2013). Research documents women's tendency to eat last and least—a phenomenon resulting from the intersections of societal norms and women's limited agency to advocate for their own needs (Gittelsohn et al., 1997). Biologic mechanisms whereby the adequate nutrition of women before, during, and after pregnancy optimizes biologic transfer of nutrition to the child in utero and early infancy contribute to improved nutritional status of the child by establishing the foundations for adequate child growth trajectories (Shrimpton et al., 2001; Victora, De Onis, Hallal, Blossner, & Shrimpton, 2010; Young et al., 2017). Additional follow-on achievements of empowerment for women's nutrition could indirectly improve child nutrition through pathways related to enhanced caring capabilities, women's self-care (Pratley, 2016), and mental health (James-Hawkins et al., 2018b; Qutteina et al., 2017; K. M. Yount, S. Dijkerman, S. Zureick-Brown, & K. E. VanderEnde, 2014).

Our work noted the importance of intrinsic agency for child nutrition. This domain comprised items related to attitudes about IPV and may reflect women's views about the right to bodily integrity (Miedema et al., 2018; Yount, VanderEnde, Dodell, & Cheong, 2016; Yount et al. under review). Hanmer and Klugman highlight attitudes about IPV as a critical domain of women's sense of empowerment, claiming that beliefs in regressive gender norms, such as IPV, minimize women's individual agency and ability to act in their own best interests. In Bangladesh, toddlers of women who reported that IPV was justified had significantly higher odds of stunting than women who rejected these attitudes. Beyond intrinsic agency, exposure to IPV negatively impacts child growth. In their review of the impacts of IPV on child nutrition, Yount, DiGirolamo, and Ramakrishnan (2011) hypothesized that IPV directly affects young children via a dysregulated stress response and indirectly via impacts on women's physical, mental, and emotional well-being.

Researchers in Kenya observed significant increases in household wealth and women's empowerment over time, including engagement in decision making and disapproval of IPV, and that wealth and these domains were positively associated with each other (Voronca et al., 2018). Our study documents effect modification of the empowerment-nutrition relationship by household wealth. Although a clear pattern failed to emerge, it is plausible that a certain level of resources is needed for women to act on their agency. For example, women may be empowered to participate in the decisions of or solely decide on how income in the household is to be used, when and how to seek care for themselves or children, or which foods to purchase for the household. If the household or community does not have financial or infrastructure resources, then the ability to act on these decisions is limited.

We recognize that the relationship between wealth and empowerment is not straightforward. Research in Bangladesh, for example, notes that women's engagement in decision making is lowest in the highest wealth groups potentially as a result of the male breadwinner having a more dominant role or the sole role in generating income (Mahmud, Shah, & Becker, 2012). Strategies that aim to empower women but are not contextualized to a community's or household's socioeconomic conditions and gender norms may inadvertently introduce inequity such that certain groups benefit while others are harmed. Thus, the efforts of government and others to mitigate poverty and economic disparity support an enabling environment in which women and households may benefit from interventions to increase agency.

Anemia in children represents a serious public health problem globally, with implications for infant development and child school performance (Allali, Brousse, Sacri, Chalumeau, & de Montalembert, 2017; Harika et al., 2017). In many countries, the prevalence of anemia exceeds the prevalence of stunting, and efforts to reduce anemia burden have been limited (Schümann & Solomons, 2017). Although studies have noted that schooling of women reduces anemia in their offspring (Harding, Aguayo, Masters, & Webb, 2018), ours is the first study, to our knowledge, to include anemia in the women's empowerment–child nutrition nexus. We noted significant and direct associations between women's instrumental agency in household decisions and child hemoglobin concentrations. Intrinsic agency (attitudes about IPV) was not directly associated with anemia. These findings contrast research in India that found that the odds of child anemia were significantly higher in households in which women experienced IPV (Ackerson & Subramanian, 2008). Differences in our findings may result from contextual differences or from differences in measurement. Although the study in India evaluated IPV experiences, our measure utilized items related to attitudes about rather personal experiences of IPV in terms of questions around the multiple scenarios where wife-beating is considered justified. Secondly, unlike other measures of nutritional status, associations between women's empowerment and anemia were not mediated through maternal nutrition. This finding suggests that effects may operate through pathways other than the biologic transfer of nutrition or the effects of a shared nutritional environment. Like stunting, the etiology of anemia is complex, with nutrition representing only one of myriad causal pathways (Ngure et al., 2014; Schümann & Solomons, 2017; Stoltzfus, 2003); a more significant pathway in some contexts involves malaria and other parasitic infections. Thus, instrumental agency may be a more relevant domain for anemia such that women have agency to seek and apply preventive and curative measures for their children.

A review of women's empowerment and child nutritional status has documented a positive association between direct and indirect measures of women's empowerment and child anthropometry (Carlson et al., 2015), although the authors note that their findings are less consistent in sub-Saharan Africa compared to other parts of the globe. Scholars argue that the contextualized nature of empowerment limits cross-context comparisons and may contribute to inconsistent findings; heterogeneity in how researchers operationalize empowerment also contributes to inconsistencies. Our work, focused on five countries in eastern sub-Saharan Africa, makes several novel contributions to this body of evidence. Other studies using DHS data operationalized women's empowerment in myriad ways, including the simple summation of responses, principle components analysis, the development of ad hoc cut-points, or use of single-item indicators as proxies (Pratley, 2016; Richardson, 2017; Yount et al., 2018). Although many of these variables are theoretically informed, they rarely are validated across contexts using systematic approaches. We used a theoretically informed approach and cross-contextually validated the invariance of our women's empowerment measure. The use of a measure that is psychometrically comparable across contexts allows for greater confidence in both cross-contextual comparisons and in the measures of association between women's empowerment and child nutrition (Miedema et al., 2018). Lastly, the application of path analysis allowed for the additional examination of maternal BMI as a key mediator that lies on the pathway between women's empowerment and child nutrition.

Despite these novel contributions, several limitations are evident in our study. Our approach was limited by those questions available in the DHS. Other measures not captured in the DHS may be salient domains of women's empowerment for child nutrition (Moghadam & Senftova, 2005) including, for example, women's time-use (Johnston, Stevano, Malapit, Hull, & Kadiyala, 2015; Njuki et al., 2016), self-efficacy, and social capital (Crandall, Rahim, & Yount, 2015; Ickes et al., 2017). Similarly, nutritional outcomes such as BMI and anemia have complex etiologies, including dietary patterns, disease exposure, and access to general and reproductive health services. We attempted to include other indicators as mediators in the path analysis related to child diets (diet diversity, minimum acceptable diets); however, the categorical nature of these data and limited variation in responses prevented convergence and reduced model fit. Further, cross-sectional survey data limits our ability to test how resources for empowerment may influence women's agency and then nutritional status (Kishor & Subaiya, 2008). The use of cross-sectional data fails to capture the inherent nature of empowerment as a process. Finally, the four-year range across surveys may result in bias due to unforeseeable events that occurred across settings during the duration of data collection. Because of data limitations, we also were unable to include Burundi, another East African country with DHS data. Although we focused specifically on East Africa, we recommend replication of this analysis in other regions and cross-regionally to assess direct and indirect effects of women's empowerment on individual health across multiple contexts.

## 5 Conclusion

Using structural equation modeling, we observed that a previously developed, three-domain latent construct of women's empowerment grounded in Kabeer's assets, agency, and achievements framework functioned through maternal BMI to influence child growth indicators but not anemia. The paths of influence from empowerment to child nutrition indicators differed by household wealth status. These findings suggest that to achieve impacts on child nutrition, designers of empowerment strategies should consider a strategy's potential to affect change in women's nutrition and be cognizant of the resource constraints in the household or community.

## Contributions

Rebecca Jones, Usha Ramakrishnan, and Amy Webb Girard conceptualized the research question. Rebecca Jones, Regine Haardörfer, Amy Webb Girard, Usha Ramakrishnan and Kathryn Yount conceptualized the analysis plan; Regine Haardörfer and Kathryn Yount guided analysis. Stephanie Miedema constructed the empowerment measure. All authors contributed to interpretation of results. Rebecca Jones and Amy Webb Girard wrote the first and subsequent drafts of the article. All authors contributed to critically revising the article and gave final approval of the version to be published.

## Statement of ethics

This secondary analysis was conducted using Demographic and Health Survey data, for which the protocol was reviewed and approved by the ethics committee for each individual country. Written informed consent was obtained from all study participants before interview. The analysis presented here used deidentified secondary data, and additional human subjects review was not required.

## Declaration of competing interest

The authors declare they have no conflicts of interest.

## Footnotes

1 Nutrition-specific interventions aim to directly improve nutrient intakes and diet practices; examples include diet-related behavior change, supplementation, and fortification. Research documents that, on their own, they are insufficient to achieve target reductions in stunting even when delivered at scale and with high (>80%) coverage (Black, Victora, Walker, Bhutta, Christian, De Onis, & al., 2013; Ruel & Alderman, 2013).

2 Human Development Index is a summary measure of average achievement in key dimensions of human development: a long and healthy life, being knowledgeable, and having a decent standard of living. Website: http://hdr.org/en/content/human-development-index.hdi.

3 Education, commonly considered an enabling resource in Kabeer's framework and participation in economic activities were each tested in EFA but dropped in subsequent analyses due to inconsistent and low factor loadings across countries.
