# Supplementary material for: Women's empowerment and child nutrition: The role of intrinsic agency
Source: SSM Popul Health. 2019 Nov 20;9:100475. doi: 10.1016/j.ssmph.2019.100475 (PMC6978483; doi:10.1016/j.ssmph.2019.100475)
Supplement: Multimedia component 1 [file mmc1.pdf]

Appendix

Supplemental Table 1. Instrument Detail for all components of Path Analysis

Domain	Survey Question	Measurement Scale
**Child Nutritional Status**		
Height for Age Z score	Standing height (> 24 mo) or recumbent length (<24 mo) measured using wooden stadiometer	Continuous (WHO Child Growth Standards)
Weight for Age Z score	Weight measured using mother-baby electronic scales	Continuous (WHO Child Growth Standards)
Anemia Status	Altitude-adjusted hemoglobin with HemoCue Photometer	Dichotomous (Anemic: < 11.0 g/dL)
**Women's Empowerment**		
Latent Factor: Human & Social Assets	How old were you when you had sexual intercourse for the very first time?	Continuous (years)
	How old were you when you first started living with [current or former partner]?	Continuous (years)
	In what month and year was [CHILD] born? (PROBE: What is his/her birthday?)	Continuous (Month/Year)
Latent Factor: Intrinsic Agency	In your opinion, is a husband justified in hitting or beating his wife in the following situations?	Dichotomous (Yes/ No / DK)
	- If she goes out without telling him?	
	- If she neglects the children?	
	- If she argues with him?	
	- If she refuses to have sex with him?	
	- If she burns the food?	

Latent Factor: Instrumental Agency	Who usually decides how the money you earn will be used?	Dichotomous (Mainly you, Mainly your husband/partner, you and your husband/partner jointly / no involvement in decision)
	Who usually makes decisions about health care for yourself?	
	Who usually makes decisions about making major household purchases?	
	Who usually makes decisions about visits to your family or relatives?	
**Mediator**		
Women's BMI	Directly measured height and weight	Continuous (Body Mass Index (kg/m2))

[a] Adapted from Table 6 of Miedema, S. S., Haardörfer, R., Girard, A. W., & Yount, K. M. (2018). Women’s Empowerment in East Africa: Development of a cross-country comparable measure. *World Development, 110*, 453-464. doi:10.1016/j.worlddev.2018.05.031

Supplemental Table 2. Selected Characteristics of east African women and children sampled by Demographic Health Surveys (2011-2016) stratified by country (n=13,780)

Variable	Ethiopia 2011 (n=2664) Mean / %	SD	Kenya 2014 (n=4918) Mean / %	SD	Rwanda 2014-15 (n=1925) Mean / %	SD	Tanzania 2015-16 (n=2490) Mean / %	SD	Uganda 2011 (n=1783) Mean / %	SD
**Domain: Human/Social Assets**										
Age at first sex	16.6	345	17.0	3.27	20.5	3.62	16.9	2.78	16.5	2.73
Age at first cohabitation	16.8	3.68	18.7	3.83	21.3	3.61	18.6	3.57	17.7	3.43
Age at first birth	19.0	3.68	19.4	3.61	22.0	3.65	19.4	3.35	18.5	3.24
**Domain: Intrinsic Agency**										
Goes out without spouse	50%		69%		79%		56%		63%	
if neglects child	43%		56%		72%		50%		52%	
if argues with husband	47%		71%		80%		56%		70%	
if refuses sex	53%		77%		75%		67%		77%	
if burns food	49%		89%		92%		79%		84%	
**Domain: Instrumental Agency**										
for respondent's earnings	89%		89%		87%		89%		83%	
for respondent's health	72%		72%		85%		71%		64%	
for large purchases	64%		65%		73%		41%		60%	
for family/friends visits	74%		67%		85%		58%		61%	
**Mediator:**										
Women's BMI	20.0	2.89	22.7	4.27	23.1	3.49	22.9	4.14	22.1	3.63
**Outcomes: Child Nutritional Status**										
Height for Age Z Score	-1.3	1.80	-1.0	1.48	-1.4	1.47	-1.3	1.43	-1.2	1.61
Weight for Age Z Score	-0.9	1.28	-0.1	1.24	0.4	1.27	-0.2	1.25	-0.6	1.26
Anemia Status	35%				49%		25%		35%	

[a] Abbreviation: SD- Standard Deviation

Supplemental Figure 1. Diagram of Direct and Indirect Standardized Path Coefficients from Women's Empowerment to Child's Nutritional Status by Country

A. Ethiopia (n=2664)

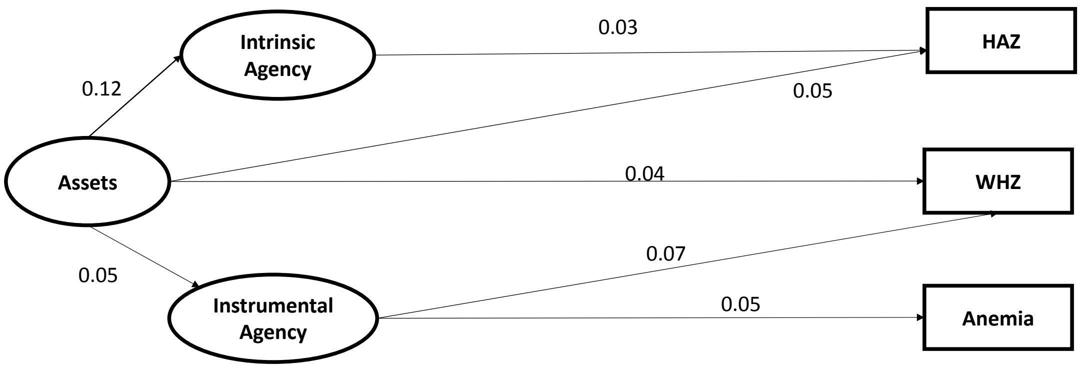

Note:

[a] Model fit statistics: df=78; CFI=0.994; TLI: 0.992; RMSEA: 0.014 (0.011,0.017)

[b] Significant paths based upon 95% confidence intervals

[c] All non-significant paths were dropped for simplicity

[d] Abbreviations: HAZ- Child height-for-age-z score; WHZ- Child weight-for-age-z score

B. Kenya (n=4918)

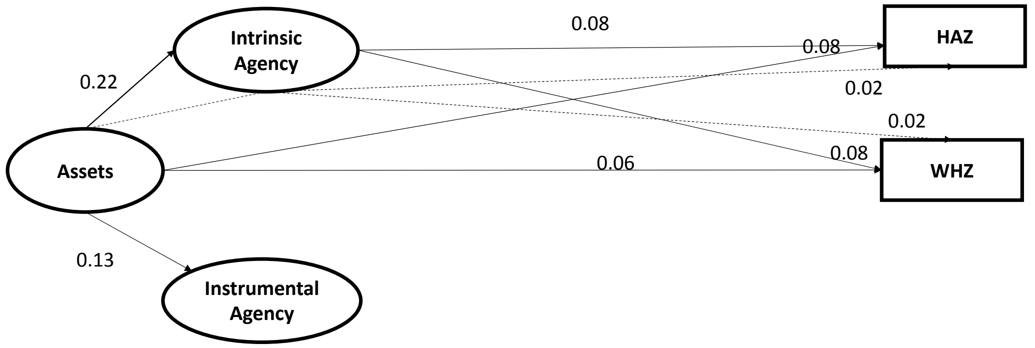

Note:
[a] Model fit statistics: df=69; CFI=0.993; TLI: 0.991; RMSEA: 0.016 (0.013,0.019)
[b] Significant paths based upon 95% confidence intervals
[c] All non-significant paths were dropped for simplicity
[d] Abbreviations: HAZ- Child height-for-age-z score; WHZ- Child weight-for-age-z score

C. Rwanda (n=1925) – no significant associations

D. Tanzania (n=2490)

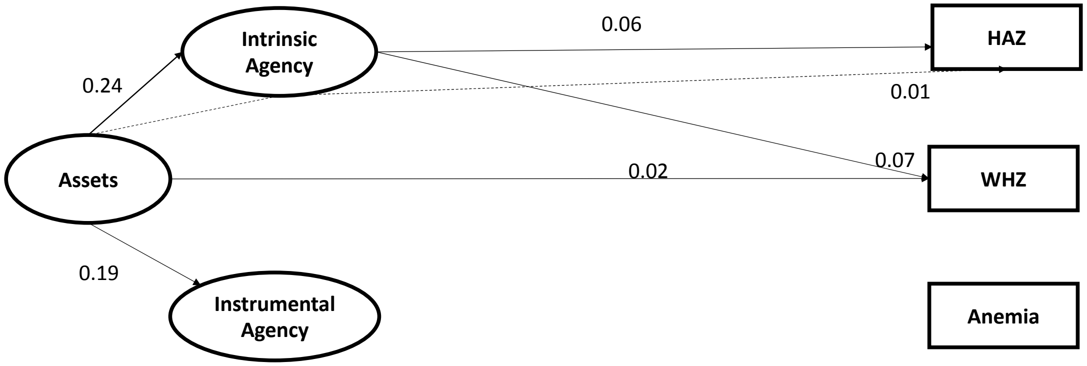

Note:
[a] Model fit statistics: df=78; CFI=0.987; TLI: 0.983; RMSEA: 0.025 (0.022,0.027)
[b] Significant paths based upon 95% confidence intervals
[c] All non-significant paths were dropped for simplicity
[d] Abbreviations: HAZ- Child height-for-age-z score; WHZ- Child weight-for-age-z score

E. Uganda (n=1783)

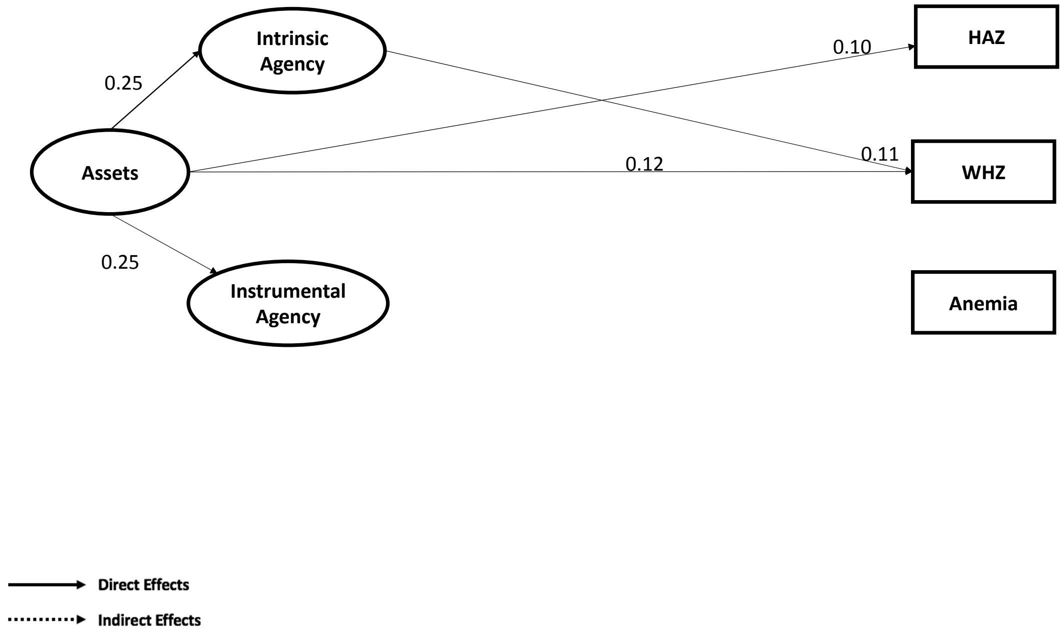

Note:
[a] Model fit statistics: df=78; CFI=0.988; TLI: 0.984; RMSEA: 0.017 (0.008,0.025)
[b] Significant paths based upon 95% confidence intervals
[c] All non-significant paths were dropped for simplicity
[d] Abbreviations: HAZ- Child height-for-age-z score; WHZ- Child weight-for-age-z score

Supplemental Figure 2. Diagram of Direct and Indirect Standardized Path Coefficients from Women's Empowerment to Child's Nutritional Status Mediated by Women's BMI by Country

A. Ethiopia (n=2664)

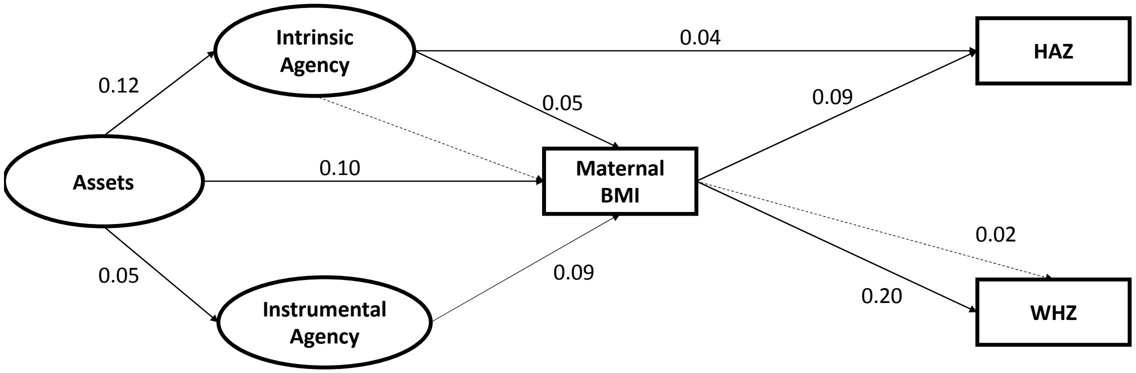

Note:

[a] Model fit statistics: df=87; CFI=0.994; TLI: 0.991; RMSEA: 0.014 (0.011,0.017)

[b] Significant paths based upon 95% confidence intervals

[c] All non-significant paths were dropped for simplicity

[d] Abbreviations: BMI- Body Mass Index; HAZ- Child height-for-age-z score; WHZ- Child weight-for-age-z score

B. Kenya (n=4918)

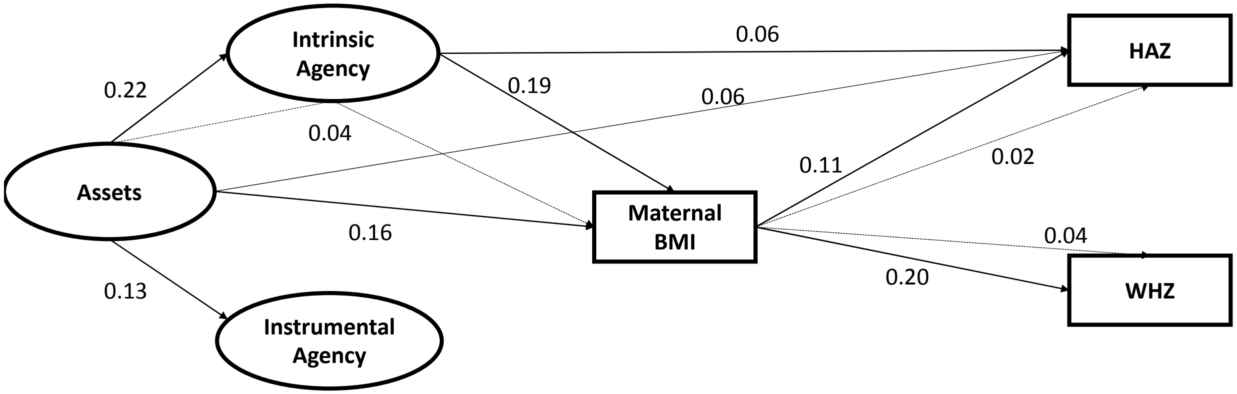

Note:

[a] Model fit statistics: df=78; CFI=0.993; TLI: 0.990; RMSEA: 0.016 (0.013,0.019)

[b] Significant paths based upon 95% confidence intervals

[c] All non-significant paths were dropped for simplicity

[d] Abbreviations: BMI- Body Mass Index; HAZ- Child height-for-age-z score; WHZ- Child weight-for-age-z score

<u>C. Rwanda (n=1925)</u>

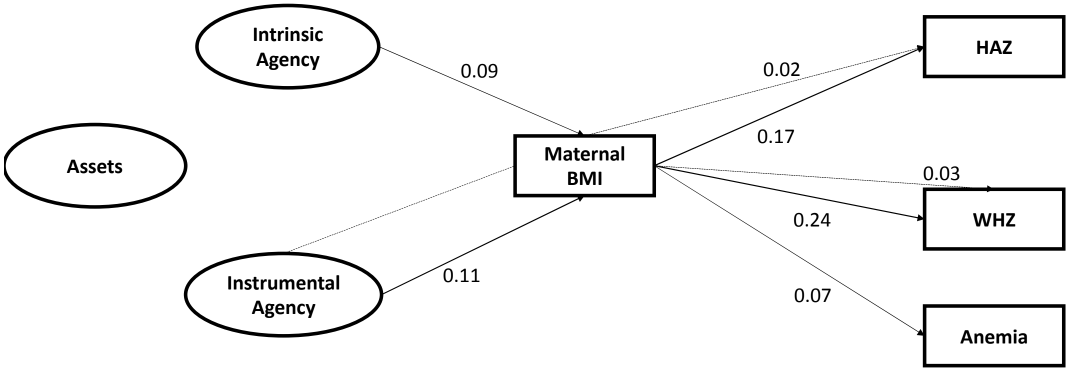

Note:
[a] Model fit statistics: df=87; CFI=0.995; TLI: 0.993; RMSEA: 0.013 (0.002,0.020)
[b] Significant paths based upon 95% confidence intervals
[c] All non-significant paths were dropped for simplicity
[d] Abbreviations: BMI- Body Mass Index; HAZ- Child height-for-age-z score; WHZ- Child weight-for-age-z score

D. Tanzania (n=2490)

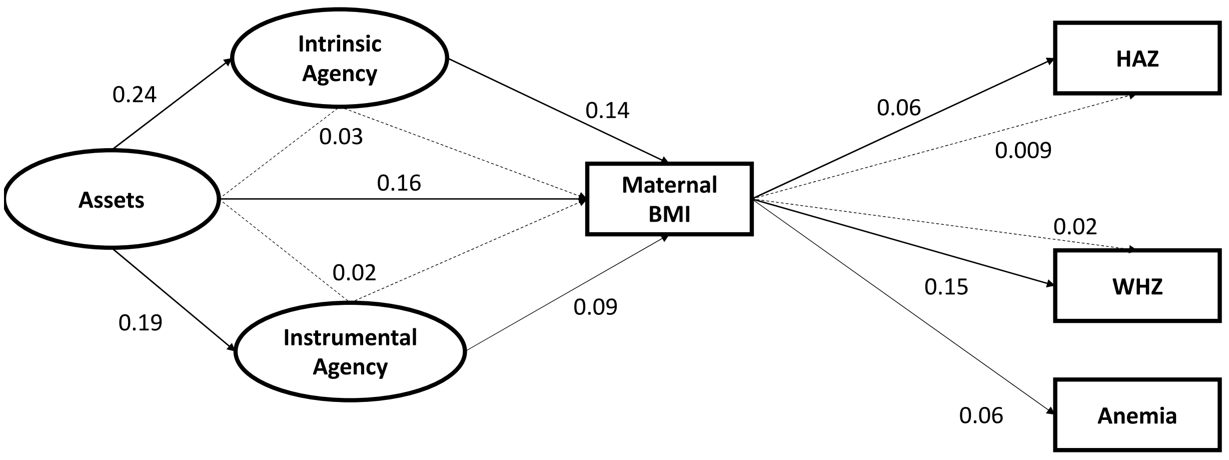

Note:

[a] Model fit statistics: df=87; CFI=0.986; TLI: 0.981; RMSEA: 0.024 (0.021,0.027)

[b] Significant paths based upon 95% confidence intervals

[c] All non-significant paths were dropped for simplicity

[d] Abbreviations: BMI- Body Mass Index; HAZ- Child height-for-age-z score; WHZ- Child weight-for-age-z score

<u>E. Uganda (n=1783)</u>

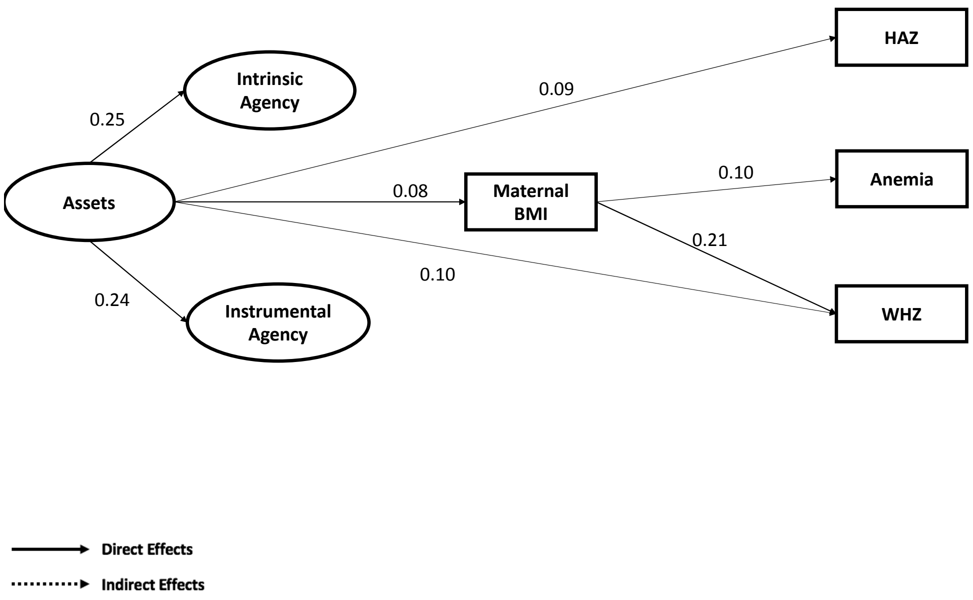

Note:
[a] Model fit statistics: df=87; CFI=0.987; TLI: 0.982; RMSEA: 0.018 (0.009,0.025)
[b] Significant paths based upon 95% confidence intervals
[c] All non-significant paths were dropped for simplicity
[d] Abbreviations: BMI- Body Mass Index; HAZ- Child height-for-age-z score; WHZ- Child weight-for-age-z score

Supplemental Figure 3. Diagram of Direct and Indirect Standardized Path Coefficients from Women's Empowerment to Child's Nutritional Status Mediated by Women's BMI by Wealth Groups and by Country

A. Ethiopia (n=2664)

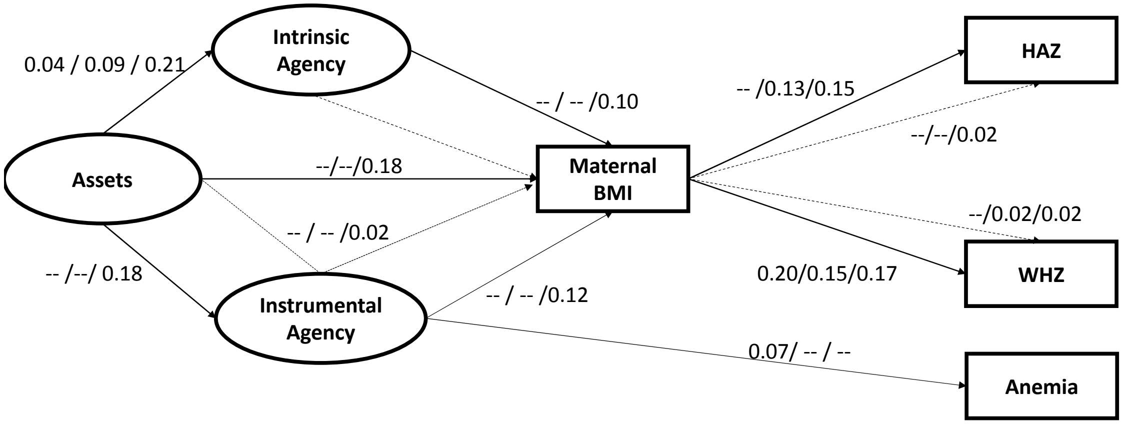

Note:

[a] Model fit statistics: df=285; CFI=0.988; TLI: 0.985; RMSEA: 0.003 (0.027,0.032)

[b] Low / Middle / High Wealth Group

[c] Significant paths based upon 95% confidence intervals

[d] All non-significant paths were dropped for simplicity

[e] Abbreviations: BMI- Body Mass Index; HAZ- Child height-for-age-z score; WHZ- Child weight-for-age-z score

B. Kenya (n=4918)

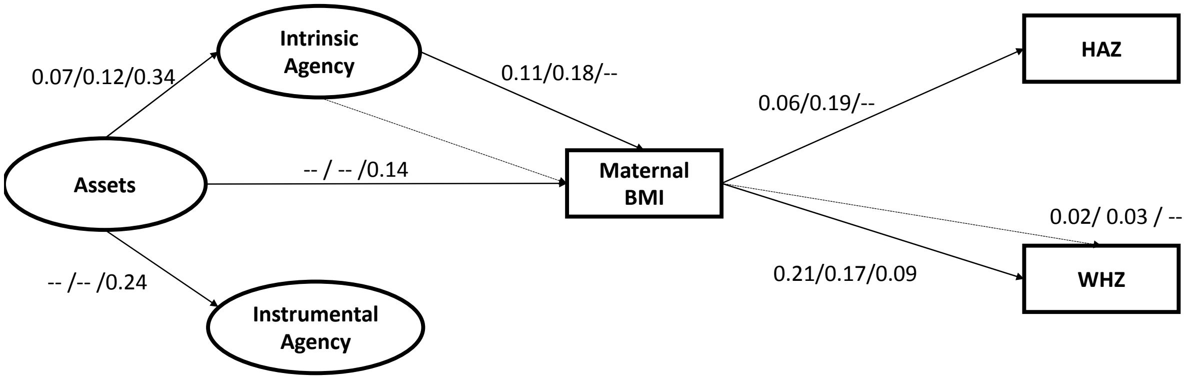

Note:
[a] Model fit statistics: df=258; CFI=0.972; TLI: 0.966; RMSEA: 0.030 (0.028,0.033)
[b] Low / Middle / High Wealth Group
[c] Significant paths based upon 95% confidence intervals
[d] All non-significant paths were dropped for simplicity
[d] Abbreviations: BMI- Body Mass Index; HAZ- Child height-for-age-z score; WHZ- Child weight-for-age-z score

C. Rwanda (n=1925)

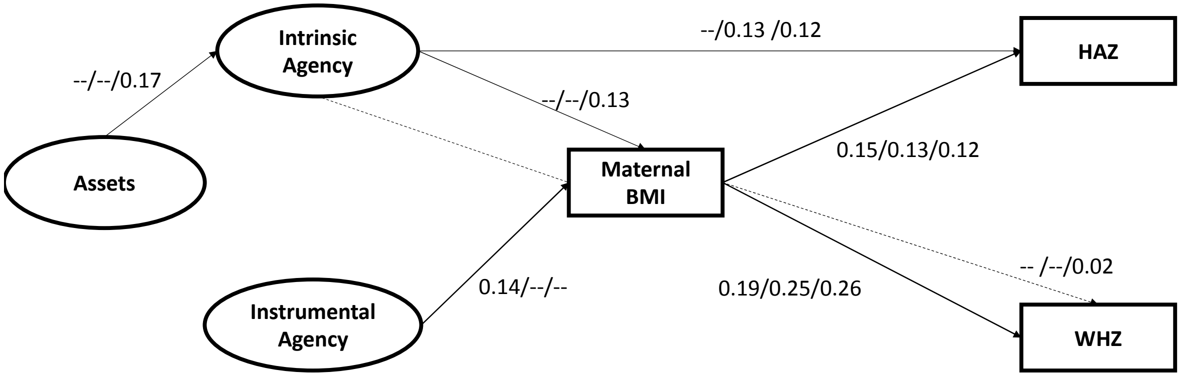

Note:
[a] Model fit statistics: df=285; CFI=0.970; TLI: 0.962; RMSEA: 0.044 (0.039,0.050)
[b] Low / Middle / High Wealth Group
[c] Significant paths based upon 95% confidence intervals
[d] All non-significant paths were dropped for simplicity
[e] Abbreviations: BMI- Body Mass Index; HAZ- Child height-for-age-z score; WHZ- Child weight-for-age-z score

D. Tanzania (n=2490)

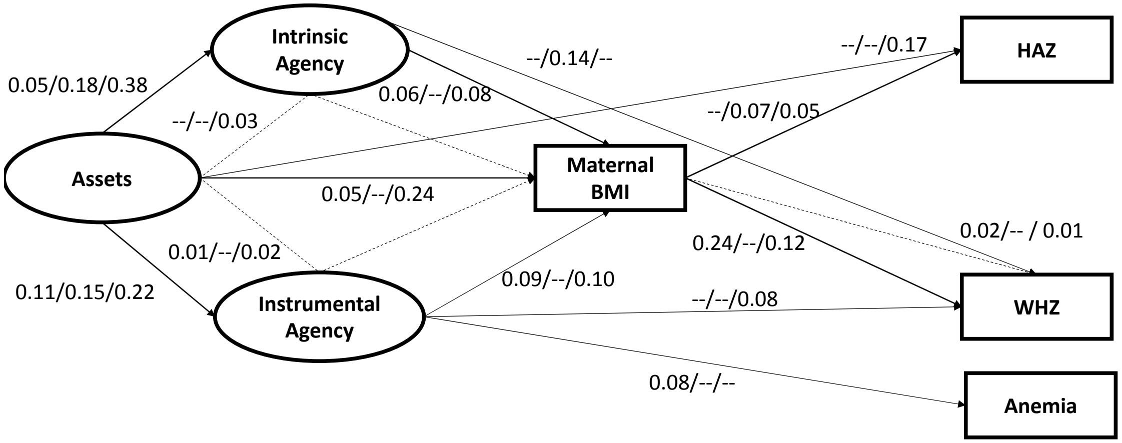

Note:
[a] Model fit statistics: df=285; CFI=0.967; TLI: 0.958; RMSEA: 0.057 (0.054,0.059)
[b] Low / Middle / High Wealth Group
[c] Significant paths based upon 95% confidence intervals
[d] All non-significant paths were dropped for simplicity
[e] Abbreviations: BMI- Body Mass Index; HAZ- Child height-for-age-z score; WHZ- Child weight-for-age-z score

<u>E. Uganda (n=1783)</u>

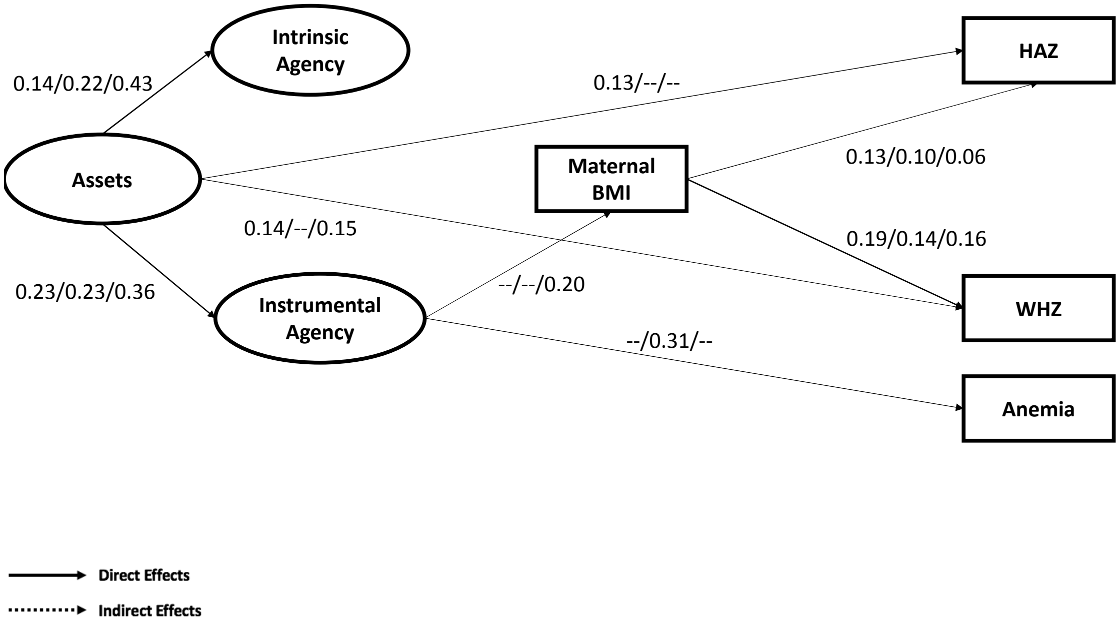

Note:
[a] Model fit statistics: df=285; CFI=0.941; TLI: 0.925; RMSEA: 0.054 (0.048,0.060)
[b] Low / Middle / High Wealth Group
[c] Significant paths based upon 95% confidence intervals
[d] All non-significant paths were dropped for simplicity
[e] Abbreviations: BMI- Body Mass Index; HAZ- Child height-for-age-z score; WHZ- Child weight-for-age-z score

Supplemental Figure 4. Diagram of Direct and Indirect Standardized Path Coefficients from Women's Empowerment to Child's Nutritional Status Mediated by Women's BMI by Wealth Groups Excluding Rwanda

A. Low

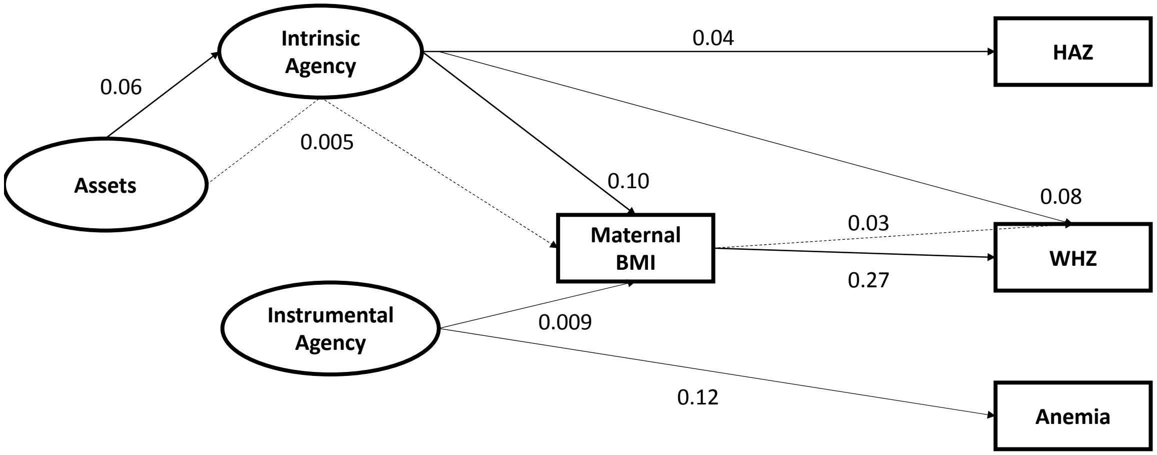

B. Middle

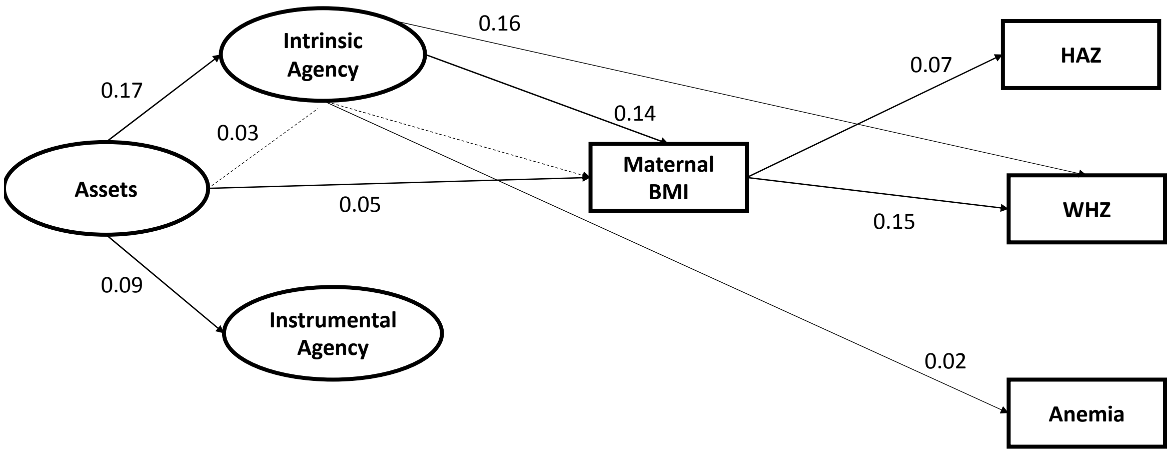
Intrinsic Agency
0.16
0.17
0.03
0.14
0.07
HAZ
Assets
0.05
Maternal BMI
0.15
WHZ
0.09
Instrumental Agency
0.02
Anemia

C. High

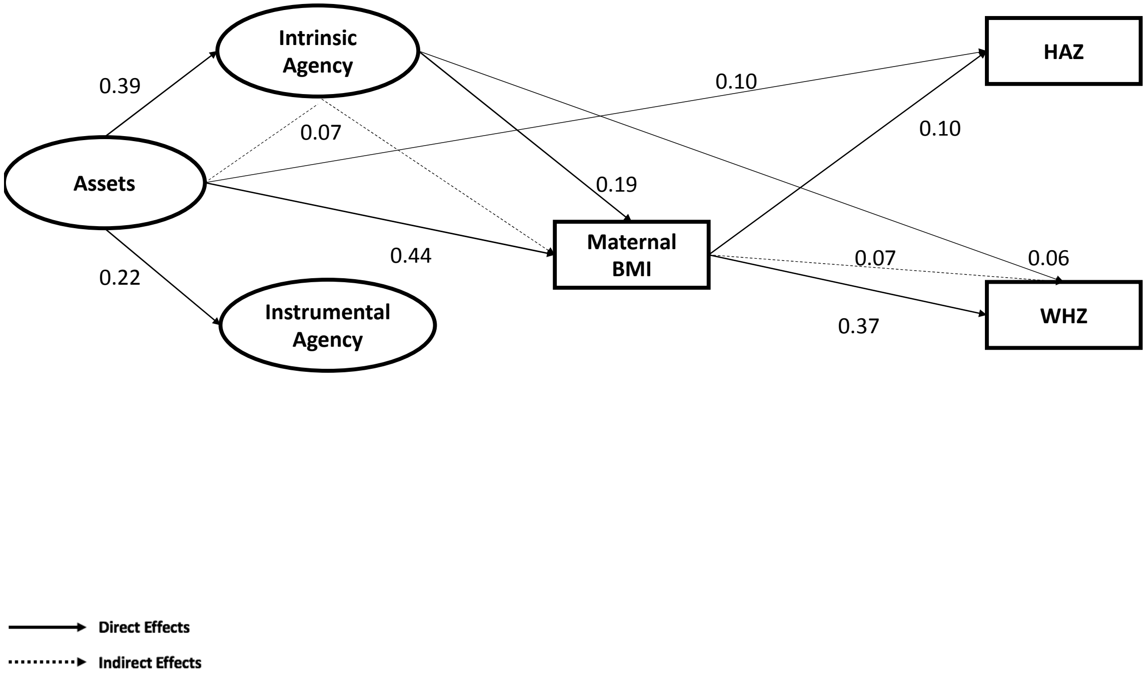

Note:
[a] Model fit statistics: df=285; CFI=0.989; TLI: 0.986; RMSEA: 0.001 (0.008,0.012)
[b] Significant paths based upon 95% confidence intervals
[c] All non-significant paths were dropped for simplicity
[d] Abbreviations: BMI- Body Mass Index; HAZ- Child height-for-age-z score; WHZ- Child weight-for-age-z score
